# Antibacterial Activity and Action Mechanism of Bacteriocin Paracin wx7 as a Selective Biopreservative against Vancomycin-Resistant *Enterococcus faecalis* in Fresh-Cut Lettuce

**DOI:** 10.3390/foods13101448

**Published:** 2024-05-08

**Authors:** Qian Zhao, Qingling Zhao, Jiabo Li, Lanhua Yi

**Affiliations:** 1 College of Food Science, Southwest University, Chongqing 400715, China; 2 Research Center for Fruits and Vegetables Logistics Preservation and Nutritional Quality Control, Southwest University, Chongqing 400715, China; 3 Chongqing Key Laboratory of Speciality Food Co-Built by Sichuan and Chongqing, Southwest University, Chongqing 400715, China

**Keywords:** biopreservative, fresh-cut lettuce, vancomycin-resistant *E. faecalis*, bacteriocin, action mechanism

## Abstract

Fresh-cut vegetables are widely consumed, but there is no food preservative available to selectively inhibit vancomycin-resistant *E. faecalis*, which is a serious health menace in fresh-cut vegetables. To develop a promising food biopreservative, a bacteriocin, paracin wx7, was synthesized, showing selective inhibition against *E. faecalis* with MIC values of 4–8 μM. It showed instant bactericidal mode within 1 h at high concentrations with concomitant cell lysis against vancomycin-resistant *E. faecalis*. Its lethal effect was visualized in a dose-dependent manner by PI/SYTO9 staining observation. The results of an in vivo control experiment carried out on *E. faecalis* in fresh-cut lettuce showed that 99.97% of vancomycin-resistant *E. faecalis* were dead after 64 μM paracin wx7 treatment for 7 days without influencing total bacteria. Further, the action mechanism of paracin wx7 was investigated. Confocal microscopy showed that paracin wx7 was located both on the cell envelope and in cytoplasm. For the cell envelope, the studies of membrane permeability using SYTOX Green dyeing and DNA leakage revealed that paracin wx7 damaged the membrane integrity of *E. faecalis*. Simultaneously, it exhibited membrane depolarization after analysis using DiSC_3_(5). Damage to the cell envelope resulted in cell deformation observed by scanning electron microscopy. On entering the cytoplasm, the paracin wx7 induced the production of endogenous reactive oxygen species.

## 1. Introduction

The consumption of fresh-cut vegetables is popular with consumers as it can provide more nutrition than their consumption after cooking [1], especially in terms of vitamin, polyphenol content, etc. However, an increasing number of antibiotic-resistant *E. faecalis* have been detected in fresh agricultural produce [2,3]. *E. faecalis* is an opportunistic pathogen, accounting for 85–90% of Enterococcal infections. It can cause life-threatening infections, such as bacteremia, endocarditis, sepsis, meningitis, surgical wound infections, and intra-abdominal infections [4,5]. Up to now, a large number of genes resistant to ampicillin, gentamicin, cephalothin, ofloxacin, teicoplanin, tetracycline, vancomycin, linezolid, daptomycin, etc., have been found among the *E. faecalis* strains [6,7,8,9]. Antibiotic resistance has made *E. faecalis* more dangerous. Among these, vancomycin-resistant *E. faecalis* has become a true menace to communal health because the ineffectiveness of the “last resort antibiotic” leads to a particular challenge for clinical management. Lettuce is one of the most consumed fresh-cut vegetables and has become an important source of vancomycin-resistant *E. faecalis* [10,11]. The consumption of fresh-cut lettuce without cooking may lead to the ingestion of live vancomycin-resistant *E. faecalis*, which would threaten human health. Therefore, to ensure the food safety of fresh-cut lettuce, food preservatives for controlling vancomycin-resistant *E. faecalis* are needed.

Food preservatives are commonly used to inhibit the growth of bacteria in food. Currently, chemical preservatives are widely used, such as benzoates, sorbates, nitrites, sulfites, citric acid, and so on [12]. However, many of these chemical preservatives have proven to be health hazards, causing diseases such as skin rashes, allergies, gastrointestinal upsets, heart palpitation, breathing difficulty, brain damage, cancer, etc. [12,13]. Moreover, these chemical preservatives are only active at high concentrations [14] and many of them are not permitted in fresh-cut vegetables. Compared with chemical preservatives, food biopreservatives are preferred for their higher safety. With the deepening understanding of intestinal microflora, there are some beneficial microorganisms in natural vegetable microbial flora [15]. The consumption of fresh-cut lettuce can also provide some safe active microorganisms [16]. Therefore, targeted control is the best food safety control strategy. Namely, only the targeted pathogen is inhibited by the preservative without damaging the other natural microbiota of the vegetables. However, there is no such selective food biopreservative available in food with antibacterial activity only against *E. faecalis*.

Bacteriocins are antibacterial polypeptides which are synthesized on ribosomes. Some bacteriocins have been reported to have narrow antibacterial activity, such as thuricin CD [17] and bacteriocin ST91KM [18]. Currently, bacteriocin is one of the most widely studied food biopreservatives [19,20]. We deduce whether there are some bacteriocins which have selective antibacterial activity against *E. faecalis*. A bacteriocin, paracin wx7, was identified from the genomic DNA of lactic acid bacteria in the NCBI database (WP_003577835) in our previous study. In this work, the antimicrobial spectrum of bacteriocin paracin wx7 was studied after chemical synthesis. This study aimed to investigate the antibacterial activity of paracin wx7 against vancomycin-resistant *E. faecalis*; then, to disclose the bactericidal mechanisms by which it provides a targeted biocontrol agent to control vancomycin-resistant *E. faecalis* in fresh-cut vegetables.

## 2. Materials and Methods

### 2.1. Bacteriocin Synthesis and Antimicrobial Spectrum Measurement

The mature peptide of novel bacteriocin paracin wx7 (AFWQGIGRWLDQHFGW-NH_2_) was synthesized according to the solid-phase peptide synthesis method on a peptide synthesizer (CEM Liberty Blue, Charlotte, NC, USA) as per our previous study [21]. Briefly, 20% piperidine was used to deprotect Fmoc group and HBTU was added to activate the carboxylic acid group to form a stabilized HOBt leaving group. Then, a peptide linkage was formed between the activated carboxyl group and the amino group of the previous amino acid. Then, the bacteriocin paracin wx7 was purified by HPLC. For HPLC analysis, a column SHIMADZU Inertsil ODS-SP (4.6 × 250 mm, 5 μm) was used and peptide was detected under 220 nm. The sample was eluted at a flow rate of 1 mL/min with solvent A (0.1% trifluoroacetic in 100% water) and solvent B (0.1% trifluoroacetic in 100% acetonitrile).

The antimicrobial spectrum of bacteriocin paracin wx7 was measured according to the value of minimal inhibitory concentration (MIC), which was conducted using the micro-broth dilution method [22]. Briefly, paracin wx7 was diluted in MH broth with concentrations from 64 μM to 0.5 μM in a 96-well plate. Subsequently, cell suspension indicator was added into wells with a final concentration of about 10^6^ CFU/mL. The plates were incubated at 37 °C for 18–24 h before result observation. The pathogen information is shown in Table 1, including typical strains and foodborne strains. The 6 foodborne *E. faecalis* strains were isolated from fresh-cut fruits. TSB broth was used for *E. faecalis*, MH broth was used for the others. In addition, the MIC value of nisin (Sigma, Phoenix, AZ, USA) to *E. faecalis* ATCC51575 was also measured.

For a better repeatability for other researchers, the typical vancomycin-resistant strain *E. faecalis* ATCC51575 was used as the indicator in the following studies.

### 2.2. Growth Curve and Time-Kill Curve

The effects of paracin wx7 on the growth and survival of *E. faecalis* were analyzed as in our previous study [23] with some modifications. For growth, *E. faecalis* was incubated under 250 rpm to logarithmic phase (OD_600nm_ = 0.3) at 37 °C. Then, 0.5, 1, 2, and 4 × MIC paracin wx7 dosages were added with three replicates. The OD_600nm_ value was measured.

For survival, log-phase cells of *E. faecalis* were collected and resuspended in sterile saline with OD_600nm_ = 0.2. Then, 2, 4 and 8 × MIC paracin wx7 was added with three replicates. Subsequently, cells were incubated at 37 °C under 250 rpm for 5 h. During this time, samples were taken out every hour, and then diluted and spread on TSA agar plates. The colonies were counted after incubation at 37 °C for 24 h. The same treatments with sterile water and 64 μM nisin were used as a negative control and a positive control, respectively, for both growth and survival assays.

### 2.3. Selective Control of E. faecalis in Fresh-Cut Lettuce

Fresh lettuce was obtained from a local supermarket in Beibei district, Chongqing, China. Referring to Woo, et al. [24], the lettuce was cut into pieces (4–6 cm × 4–6 cm) after washing using running water. *E. faecalis* was prepared in sterile saline around cell density of 10^7^ CFU/mL. The fresh-cut lettuce was dipped into the *E. faecalis* suspension for 5 min, and then dried under room temperature for 2 h. The fresh-cut lettuce inoculated with *E. faecalis* was packaged in boxes (about 100 g per box), and then 10 mL 64 μM paracin wx7 or sterile saline (control) was sprayed evenly over the fresh-cut lettuce. Each treatment had four replicates (4 boxes), three were used for microorganism count analysis, and the other one was used to observe the sensory quality. Each day, a 10 g sample was taken out to analyze the colony numbers of *E. faecalis* and total bacteria, as in our previous study [21]. For *E. faecalis*, the selective medium (*E. faecalis* Agar, Hopebio, Qingdao, China) was used. 

### 2.4. Live/Death and Cell Aggregation

Paracin wx7 was added into log-phase *E. faecalis* (OD_600nm_ = 0.2) with final concentrations of 1, 2, and 4 × MIC. Cells of *E. faecalis* were treated at 37 °C (250 rpm) for 1 h, and then washed and dyed according to the kit description of a Live/Dead BacLight Bacterial Viability kit (ThermoFisher, Waltham, MA, USA). Avoiding light for 20 min, and then cells were applied to an Eclipse Ti2 fluorescence microscope (Nikon, Tokyo, Japan) to observe fluorescence images. The same treatment of *E. faecalis* cells with sterile water instead of bacteriocin paracin wx7 was used as the control.

The effect of paracin wx7 on cell aggregation of *E. faecalis* was analyzed. *E. faecalis* was prepared in sterile saline with a cell density of OD_600nm_ = 0.68, and then 1 and 2 × MIC paracin wx7 dosages were added. Cells were treated with paracin wx7 for 1 h (37 °C, 250 rpm), followed by washing and resuspension in sterile saline. OD_600nm_ value of cell suspension at appointed times (0, 12, 24, and 36 h) was measured without vibration. Cell aggregation (%) = (1 − A*_t_*/A_0_) × 100, where A_0_ is the OD_600nm_ value at 0 h, and A*_t_* is the OD_600nm_ value at each appointed time.

### 2.5. Fluorescence Microscope and Confocal Microscope

Cell suspension of *E. faecalis* was prepared as above and then treated with FITC-labeled paracin wx7 (FITC-paracin wx7) for 1 h at 37 °C. The treatment concentrations of paracin wx7 were 1, 2, and 4 × MIC, respectively. Residual paracin wx7 was removed by washing and fluorescence of cells was observed on the Eclipse Ti2 fluorescence microscope. The same treatment of *E. faecalis* cells with sterile water instead of paracin wx7 was used as the control.

For confocal microscope analysis, cell suspension of *E. faecalis* was first stained with 10 μg/mL dye DAPI for 30 min in the dark. Cells of *E. faecalis* were washed and 2 × MIC FITC-paracin wx7 was added, followed by incubation at 37 °C for 1 h. Cells of *E. faecalis* were washed again and fixed with 2.5% glutaraldehyde (Sigma, USA), the fluorescence distribution in cells was observed on a Leica TCS SPE Confocal Microscope (Leica, Wetzlar, Germany). Blue fluorescence observation of DAPI was conducted under the 405 nm excitation and green fluorescence of FITC was conducted under the 488 nm excitation.

### 2.6. Membrane Permeability

The influence of paracin wx7 on the membrane permeability of *E. faecalis* was investigated by SYTOX Green dyeing as per our previous study [21] and DNA release. For SYTOX Green dyeing, cells with 1 μM SYTOX Green were treated with 1× MIC and 2 × MIC paracin wx7. Fluorescence strength (excitation 488 nm/emission 523 nm) was measured every 10 min for 60 min. The controls were the same treatments of *E. faecalis* cells with 16 μg/mL melittin (positive control) and sterile water (negative control). For DNA release, the cell suspensions were treated with 0.5, 1, and 2 × MIC paracin wx7 for 30 min at 37 °C, and then cell-free supernatant was collected by centrifugation. Released DNA in supernatant was measured on a NanoDrop™ One^c^ Spectrophotometer (ThermoFisher, USA). The controls were the same treatments of *E. faecalis* cells with 64 μM nisin (positive control) and sterile water (negative control).

### 2.7. Membrane Electrical Potential

A fluorescent probe DiSC_3_(5) [25] was used to measure the membrane potential of *E. faecalis*. Firstly, 100 mM KCl was added to the cell suspension of *E. faecalis*, followed by adding 1 μM DiSC_3_(5) (Sigma, USA). Cells were kept in the dark for 15 min, and then 0.5 × MIC paracin wx7 was added. Fluorescence strength of cell suspension was measured every 34 s under 620 nm excitation and 670 nm emission. The controls were the same treatments of *E. faecalis* cell suspensions with valinomycin (positive control) and sterile water (negative control). 

### 2.8. Scanning Electron Microscope (SEM) 

*E. faecalis* was incubated in TSB broth to log-phase (OD_600nm_ = 0.3). A concentration of 2 × MIC paracin wx7 was added to the cell suspension and treated for 2 h (37 °C). After washing, cells were fixed with 2.5% glutaraldehyde (Sigma, USA) overnight and dehydrated as described by Qiao et al. [26]. After drying, cells were coated with gold and subjected to scanning electron microscopy (Tescan VEGA3) to observe cell morphology.

### 2.9. Determination of Intracellular Reactive Oxygen Species (ROS) 

Referring to Sabolova et al. [27] with some modification, 2′,7′-dichlorofluorescein diacetate (DCFH-DA) was used to measure endogenous amounts of ROS in *E. faecalis*. The cell suspension of *E. faecalis* was divided into two groups. Then, 20 mM L-ascorbic acid (Sigma, USA) was added into one of the two groups. Subsequently, for both groups, 0, 0.5, 1, and 2 × MIC paracin wx7 doses were added with three replicates. Cells of *E. faecalis* were treated with paracin wx7 at 37 °C for 1 h. Cells were collected by centrifugation and resuspended in the same volume of sterile saline, followed by the addition of 10 µM DCFH-DA. Fluorescence strength (excitation 488 nm/emission 525 nm) of cell suspensions was measured after being kept in the dark for 15 min.

### 2.10. Statistical Analysis

Each assay was conducted with three biological replicates and the results were expressed as mean ± standard error. Data significance analysis was conducted by one-way ANOVA and followed by Duncan’s test with *p* < 0.05. Graphs were produced using GraphPad Prism 7.

## 3. Results 

### 3.1. Antimicrobial Spectrum of Bacteriocin Paracin wx7

**Figure 1:**
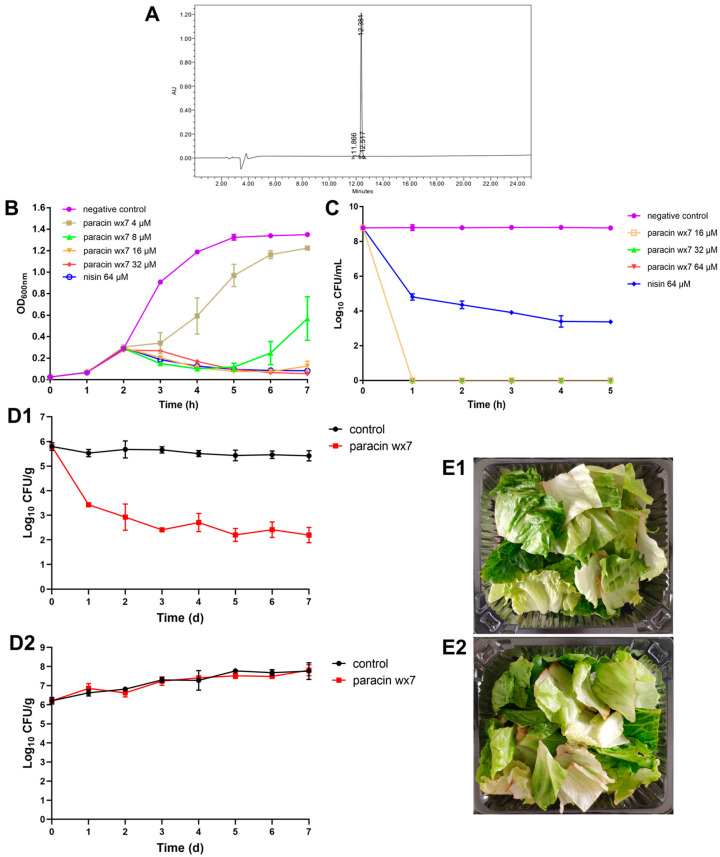
Antibacterial activity of paracin wx7 against *E. faecalis* in vitro and in fresh-cut lettuce. (**A**) Purity of paracin wx7 after HPLC; (**B**) effect of paracin wx7 on growth curve of *E. faecalis*; (**C**) effect of paracin wx7 on time-kill curve of *E. faecalis*; (**D1**) effect of paracin wx7 on vancomycin-resistant *E. faecalis*, (**D2**) effect of paracin wx7 on total bacteria in fresh-cut lettuce; (**E1**) fresh-cut lettuce with paracin wx7 treatment on the seventh day; (**E2**) fresh-cut lettuce control on the seventh day.

As shown in Figure 1A, the purity of the paracin wx7 was 98% after purification by HPLC. The results of the MIC values of paracin wx7 against 16 indicators demonstrated that paracin wx7 had no antibacterial activity against *Staphylococcus aureus*, MRSA, *Escherichia coli*, *E. coli* O157:H7, *Listeria monocytogenes*, *Salmonella*, *Pseudomonas aeruginosa* or *Klebsiella pneumoniae* (MIC values > 64 μM). However, it exhibited good antibacterial activity against eight *E. faecalis* strains with MIC values of 4 or 8 μM, including six vancomycin-resistant *E. faecalis* strains from fresh-cut fruits. The MIC value of paracin wx7 to the typical sensitive strain *E. faecalis* ATCC29212 was 4 μM, and 8 μM to the typical vancomycin-resistant strain *E. faecalis* ATCC51575. The results indicated that paracin wx7 had selective antibacterial activity against *E. faecalis* with a narrow antimicrobial spectrum. Therefore, the bacteriocin paracin wx7 could be used as a targeted antibacterial agent to control *E. faecalis*.

### 3.2. Effect of Paracin wx7 on the Growth and Survival of E. faecalis

To achieve a better understanding of the antibacterial activity of paracin wx7 against vancomycin-resistant *E. faecalis*, both the growth curve and the time-kill curve of *E. faecalis* were analyzed. For the growth curve (Figure 1B), paracin wx7 was added at a well-grown stage of the *E. faecalis* (early log-phase). The negative control maintained rapid growth and entered into a stationary phase 5 h later. The 0.5 × MIC paracin wx7 treatment delayed the growth of *E. faecalis* and ended with a lower cell density than the negative control. The 1 × MIC paracin wx7 treatment inhibited the growth of *E. faecalis* in the first 3 h, but secondary growth was observed after being treated for 4 h. The growth of *E. faecalis* was inhibited by paracin wx7 at concentrations ≥ 2 × MIC, closed to the 64 μM nisin treatment. 

The time-kill curve revealed the death speed of vancomycin-resistant *E. faecalis* after paracin wx7 treatment. As shown in Figure 1C, the viable count remained stable over the 5 h for the negative control. However, paracin wx7 rapidly killed *E. faecalis* within 1 h so that viable cells were reduced to an undetectable level at all concentrations (2 to 8 × MIC) after being treated for 1 h. For nisin, it killed *E. faecalis* in a time-dependence manner with a relatively high level of surviving cells (about 10^4^ CFU/mL). Therefore, paracin wx7 had an instant bactericidal mode to vancomycin-resistant *E. faecalis* at concentrations ≥ 2 × MIC.

### 3.3. Selective Control of E. faecalis by Paracin wx7 in Fresh-Cut Lettuce

As shown in Figure 1(D1), over a storage period of 7 days, the growth of *E. faecalis* in the control group mostly remained stable at a level of around 5.4–5.8 log_10_ CFU/mL. In contrast, *E. faecalis* in the bacteriocin-treatment group rapidly decreased to 3.4 log_10_ CFU/mL on the first day, then reduced to 2.2 log_10_ CFU/mL on the seventh day. Namely, 99.97% of the vancomycin-resistant *E. faecalis* in fresh-cut lettuce was dead after paracin wx7 treatment for 7 days. The results indicated that paracin wx7 exhibited bactericidal mode against vancomycin-resistant *E. faecalis* in fresh-cut lettuce at a concentration of 64 μM. At the same time, the total bacteria were monitored (Figure 1(D2)). With the extension of storage time, the total bacteria in both groups increased with the same tendency over the 7 days. Moreover, the total bacteria (Figure 1(D2)) and sensory quality (Figure 1(E1,E2)) in both groups had no significant differences. This result demonstrated that paracin wx7 treatment did not influence the total bacteria but reduced *E. faecalis* in fresh-cut lettuce.

### 3.4. Effect of Paracin wx7 on Live/Death and Cell Aggregation of E. faecalis

**Figure 2:**
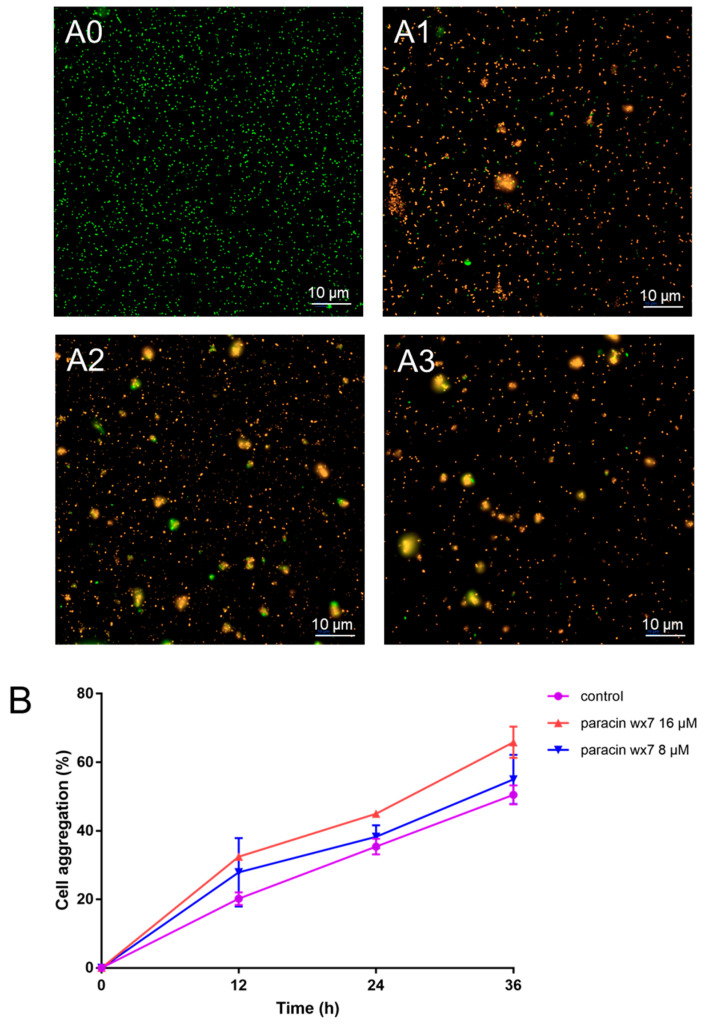
Live/death (**A**) and cell aggregation (**B**) of *E. faecalis*. (**A0**) Control; (**A1**) 1 × MIC paracin wx7 treatment; (**A2**) 2 × MIC paracin wx7 treatment; (**A3**) 4 × MIC paracin wx7 treatment.

Using two dyes, cells with compromised membranes are considered to be dead and will show red (the fluorescence of PI), whereas cells with an intact membrane will show green (the fluorescence of SYTO9) [28]. Almost all cells of *E. faecalis* in the control were green (Figure 2(A0)). However, only a low proportion of *E. faecalis* cells were green after 1 × MIC paracin wx7 treatment (Figure 2(A1)). The red cells further increased with the increase in bacteriocin concentration (Figure 2(A2,A3)). At the same time, aggregated cells were observed by fluorescence microscope after the paracin wx7 treatment. 

To further reveal whether paracin wx7 affected the quorum sensing of *E. faecalis*, cell aggregation was investigated. The results (Figure 2B) showed that paracin wx7 treatment induced cell aggregation of *E. faecalis* in a concentration-dependent manner. 

### 3.5. Action Location of Paracin wx7 

**Figure 3:**
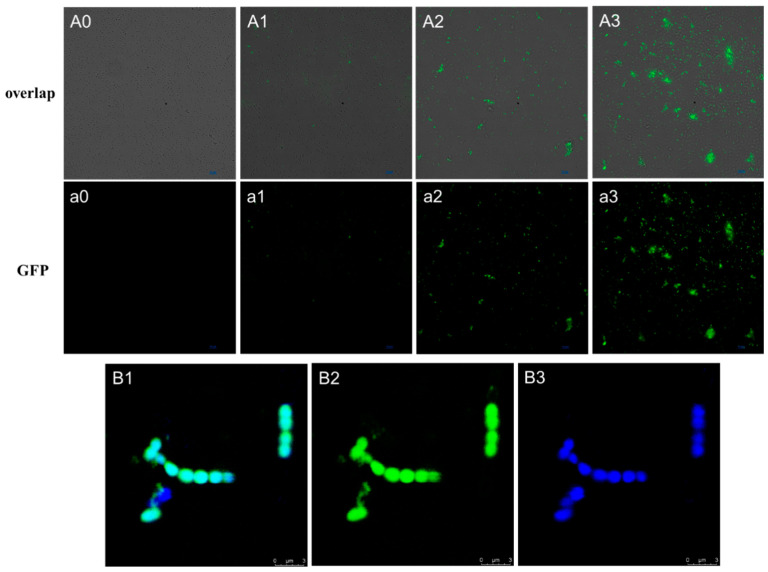
Images from fluorescence microscopy (**A**,**a**) and confocal microscopy (**B**). (**A0**,**a0**) Control; (**A1**,**a1**) 1 × MIC FITC-paracin wx7 treatment; (**A2**,**a2**) 2 × MIC FITC-paracin wx7 treatment; (**A3**,**a3**) 4 × MIC FITC-paracin wx7 treatment. (**B1**) Image of overlap; (**B2**) image of FITC-paracin wx7; (**B3**) image of DAPI.

To observe the action location of paracin wx7 on *E. faecalis*, the fluorescein FITC was labeled to paracin wx7 to trace its sites. The results of fluorescence microscopy showed that there was no fluorescence for the control (Figure 3(A0,a0)). For 1 × MIC FITC-paracin wx7 treatment, many cells were labeled with fluorescence, but the fluorescence intensity was weak (Figure 3(A1,a1)). As shown in 2 × MIC (Figure 3(A2,a2)) and 4 × MIC (Figure 3(A3,a3)) paracin wx7 treatment, an increasing number of cells were labelled with fluorescence with a concentration increase. It indicated that more paracin wx7 entered cells at high concentrations. In addition, more cells aggregated at a higher paracin wx7 concentration. 

Further, the confocal microscope was used to provide a better visualization of the location of the FITC-paracin wx7 (Figure 3B). DAPI can bind to the DNA of intact cell membranes and shows blue fluorescence under ultraviolet [29]. Fluorescence overlap between DAPI and FITC can indicate the distribution of FITC-paracin wx7. According to Figure 3(B2,B3), FITC-paracin wx7 and DAPI entered the cells. The overlap (Figure 3(B1)) showed two features: ① the edge of the cells showed stronger green fluorescence; ② the green fluorescence was uniformly distributed in the cells. This result indicated that paracin wx7 was located both on the cell envelope and in the cytoplasm.

### 3.6. Effect of Paracin wx7 on Membrane Integrity of E. faecalis

**Figure 4:**
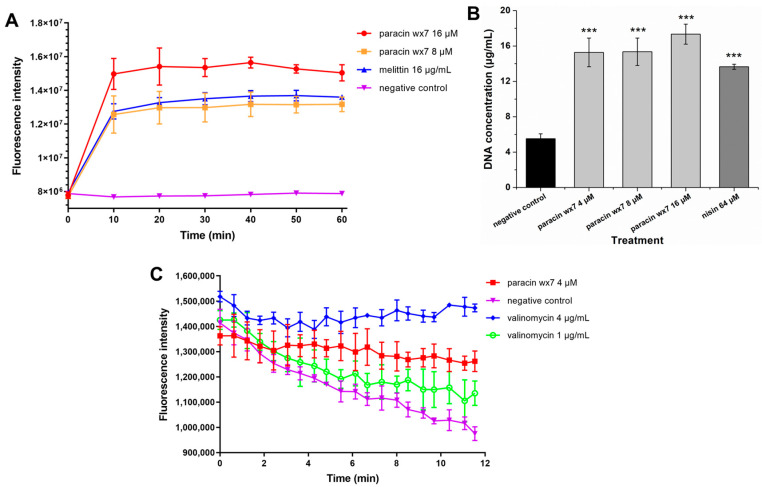
Effect of paracin wx7 on cell envelope. (**A**) Effect of paracin wx7 on membrane integrity of *E. faecalis* stained by SYTOX Green; (**B**) effect of paracin wx7 on membrane integrity by DNA release; (**C**) effect of paracin wx7 on transmembrane electrical potential in *E. faecalis*. ***: significant at *p* < 0.001.

The results of the action location indicated that some paracin wx7 existed on the cell envelope. Therefore, the membrane integrity of *E. faecalis* was first detected using SYTOX Green. SYTOX Green enters cells only when the membrane integrity is destroyed [30]. As shown in Figure 4A, the fluorescence intensity maintained a horizontal line with time for the negative control. Melittin has strong surface effects on cell membranes by forming pores and is widely used as a positive control to indicate damage to membrane integrity [31]. For 16 μg/mL melittin treatment, the fluorescence intensity rapidly increased from 7,923,997 ± 139,507 to 12,755,358 ± 458,110 within 10 min, and then slowly increased to a maximum of 13,693,871 ± 316,471 at 50 min. For 8 μM paracin wx7 treatment, the change in fluorescence intensity was close to that of 16 μg/mL melittin treatment. However, 16 μM paracin wx7 treatment induced a higher increase in fluorescence intensity. The fluorescence intensity was 14,975,311 ± 921,799 at 10 min and its maximum was 15,646,045 ± 319,251. Therefore, pore formation may also be induced by paracin wx7 treatment.

Further, DNA release was also used to indicate the damage to membrane integrity (Figure 4B). Compared with the negative control, the treatment with nisin, a pore-formation bacteriocin, significantly increased the release of intracellular DNA. At the same time, the concentrations of released DNA after being treated with paracin wx7 (4 to 16 μM) all were higher than that of 64 μM nisin. The results also demonstrated that the membrane integrity of *E. faecalis* was damaged by paracin wx7, which was in accordance with the results of SYTOX Green above.

### 3.7. Membrane Depolarization of Paracin wx7

DiSC_3_(5) is a voltage-sensitive dye and can accumulate on the polarized membranes of cells, resulting in the quenching of fluorescence. When a membrane is depolarized, the dye is released into its surroundings, which can be measured by fluorescence intensity change [32,33]. The fluorescence intensity of the negative control rapidly decreased with time (Figure 4C). For a 1 μg/mL valinomycin treatment, fluorescence quenching was relieved. For a 4 μg/mL valinomycin treatment, the fluorescence intensity was relatively stable without a huge reduction. The 0.5 × MIC paracin wx7 treatment also showed relatively stable fluorescence intensity without a huge drop. Therefore, paracin wx7 had membrane depolarization. 

### 3.8. Effect of Paracin wx7 on Cell Morphology of E. faecalis

**Figure 5:**
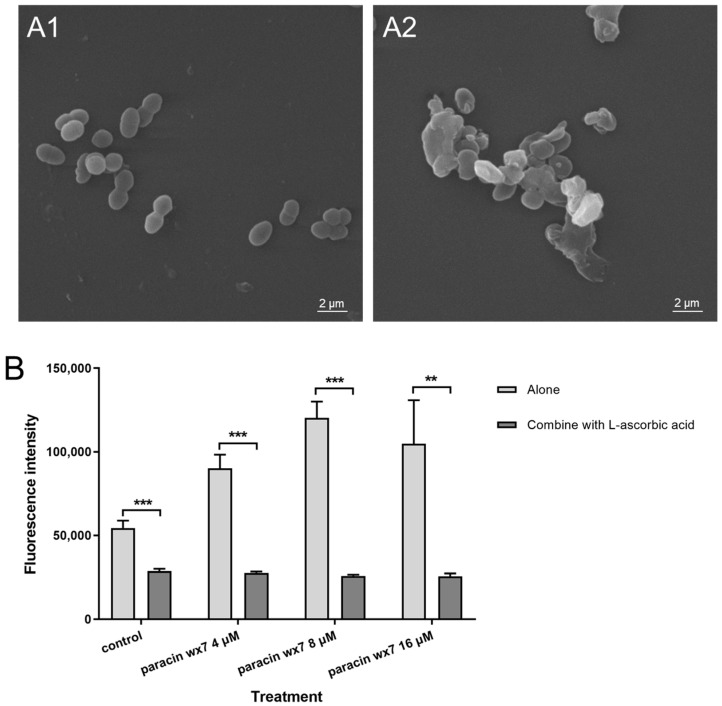
Effect of paracin wx7 on morphology (**A**) and ROS production (**B**) of *E. faecalis*. (**A1**) Cell morphology of *E. faecalis* for control, (**A2**) cell morphology of *E. faecalis* for paracin wx7 treatment. **: significant at *p* < 0.01; ***: significant at *p* < 0.001.

*E. faecalis* in the control group had an intact and plump cell profile, as well as cells distributed in pairs (Figure 5(A1)). However, after paracin wx7 treatment, there were great changes (Figure 5(A2)): ① instead of being plump, the cells had collapsed; ② instead of spheroidicity, the cell profile became irregular and deformed; ③ instead of being individual or in pairs, the envelope of the cells concatenated in aggregation. The results of both the cell surface damage for individuals and the aggregation for the population observed by SEM were in line with the results above.

### 3.9. Inducing ROS Production by Paracin wx7 

DCFH-DA was employed to monitor the production of ROS in *E. faecalis* after paracin wx7 treatment. Ascorbic acid is an excellent reducing agent, which can eliminate produced ROS. As shown in Figure 5B, both the control and paracin wx7 treatments had low levels of fluorescence intensity (without significant differences between each treatment) in the combination group containing L-ascorbic acid. In the alone group without ascorbic acid, the fluorescence intensity of paracin wx7 treatment was always higher than that of the control in a dose-dependent manner when the concentration was ≤1 × MIC. The fluorescence intensity of 2 × MIC paracin wx7 treatment was lower than that of 1 × MIC; it may be the result of the fluorescence leakage caused by great cell envelope damage. The mean absolute difference (MAD) in fluorescence intensity between the combination group and the lone group of paracin wx7 treatment (for any concentration) was greater than that of the control. Therefore, paracin wx7 treatment induced ROS production in cells of *E. faecalis*.

## 4. Discussion 

The targeted control of pathogens in vegetables without influencing the natural microbiota is the development demand for food safety control of fresh agricultural produce. Lettuce grows in soil, which implies a high contamination rate of *E. faecalis*. Vancomycin-resistant *E. faecalis* in fresh-cut lettuce draws special attention as its infection is hard to treat. In this study, the bacteriocin paracin wx7 had selective inhibition against *E. faecalis* according to the inhibition spectrum (Table 1) and the total bacteria in fresh-cut lettuce (Figure 1(D2)). The narrow antibacterial spectrum of paracin wx7 may derive from its special physicochemical properties, but more work is needed in future study to disclose the reason. The MIC values of bacteriocin paracin wx7 were 4–8 μM to both sensitive and vancomycin-resistant *E. faecalis* strains (Table 1), while that of nisin to *E. faecalis* ATCC51575 was 64 μM. This means that paracin wx7 has a much better antibacterial activity than nisin and may be a good candidate as an antimicrobial to selectively control *E. faecalis* in food, including vancomycin-resistant strains. The results of both in vitro assay (Figure 1C) and in vivo assay (Figure 1(D1)) demonstrated that paracin wx7 acted by bactericidal mode with a pathogen reduction of over 99.9% (>3 log_10_ CFU/mL) [34]. Moreover, like lactic 3147, which had a rapid killing effect against vancomycin-resistant *E. faecalis* [35], paracin wx7 showed bactericidal mode within 1 h (Figure 1C). The results of the growth curve and time-kill curve hint to us that paracin wx7 is more effective at a higher concentration. Moreover, paracin wx7 can perform better when the concentration of *E. faecalis* is below 10^8^ CFU/mL. After treatment with paracin wx7, absorbance at OD_600nm_ was significantly reduced (Figure 1B). This indicates that cell-lysis of *E. faecalis* happens during paracin wx7 treatment [36]. Rapid lysis is usually a feature of the membrane-permeabilizing action mode of antimicrobial peptides [37]. Therefore, paracin wx7 may kill *E. faecalis* by damaging the cell envelope. 

The influence of paracin wx7 on the cell envelope of *E. faecalis* was further investigated. The change in membrane integrity after paracin wx7 treatment was studied first. To analyze the membrane permeabilization ability of paracin wx7, melittin was used as the positive control as it could form big pores [38]. Excitingly, 16 μM paracin wx7 treatment had a stronger membrane permeabilization than 16 μg/mL melittin treatment (Figure 4A). Nisin also can induce pore formation by targeting lipid II and inhibiting peptidoglycan synthesis [39]. Compared with nisin, paracin wx7 showed a stronger membrane permeabilization ability (Figure 4B). These results indicate that paracin wx7 may form big pores, which may be used to damage the membrane integrity of *E. faecalis*. Subsequently, changes in membrane potential after paracin wx7 treatment were studied by DiSC_3_(5), a fluorogenic probe indicating transmembrane potential. The antibiotic valinomycin is a well-known potassium-specific transporter, which reduces the electrochemical potential gradient by facilitating the movement of potassium ions through membranes [40]. Like valinomycin, paracin wx7 also triggers the rapid loss of membrane potential (Figure 4C). Therefore, paracin wx7 may damage the cell envelope and lose the membrane potential of *E. faecalis*, ultimately, resulting in cell death. Usually, damage to the cell envelope will cause cell deformation. As expected, paracin wx7 treatment caused cell collapse with great deformation (Figure 5(A2)). All the evidence indicates that paracin wx7 damages the cell envelope of *E. faecalis*. 

The antibacterial mechanisms of bacteriocins are mainly divided into two kinds: ① damaging the cell envelope and ② inhibiting gene expression and protein production within the cell [39]. To further verify the antibacterial mechanism of paracin wx7, paracin wx7 was labeled by fluorescein FITC to trace its location in *E. faecalis*. The results of the fluorescence microscopy (Figure 3A,a) demonstrated that more paracin wx7 entered cells of *E. faecalis* with the increase in treatment concentration. Paracin wx7 was not only located in the cell envelope but also distributed in the cytoplasm within the cells (Figure 3B). There are two possibilities. One possibility is that the cell envelope is the only target, and paracin wx7 distributes in the cytoplasm just because of physical diffusion when the pore channels are available. Another possibility is that there are targets within the cells besides the cell envelope. However, paracin wx7 neither binds to DNA nor causes DNA degradation (Figure S1). In the cells, it was found that paracin wx7 treatment could induce the production of ROS. Inducing the production of endogenous ROS has been considered to be important for antibacterial activity [41]. Inducing ROS accumulation may be an important antibacterial mechanism of paracin wx7 after it enters the cytoplasm of *E. faecalis*.

## 5. Conclusions 

Currently, there is no food preservative available to selectively control *E. faecalis* in fresh-cut vegetables; vancomycin-resistance has made *E. faecalis* more dangerous. Bacteriocins from lactic acid bacteria have GRAS-safe levels and are promising food biopreservatives. In this study, the bacteriocin paracin wx7 exhibited a narrow antibacterial spectrum against *E. faecalis* alone amongst the tested pathogens. Moreover, it had very good antibacterial activity, which was close to some antibiotics, and far more effective than nisin. Paracin wx7 had a rapid bactericidal activity with concomitant cell lysis. Its action location included the cell envelope and cytoplasm. For the cell envelope, it facilitated the formation of pores in the cell membrane, resulting in the loss of membrane potential. The strong effect on the cell envelope also caused great cell deformation. For intracellular action, paracin wx7 induced the production of endogenous ROS. Namely, paracin wx7 had bactericidal action against vancomycin-resistant *E. faecalis* by cell envelope damage and inducing ROS production both in vitro and in fresh-cut lettuce. In conclusion, paracin wx7 showed great potential as a food biopreservative to selectively control vancomycin-resistant *E. faecalis* in fresh-cut vegetables.

## Figures and Tables

**Table 1:** MIC values of bacteriocin paracin wx7 against pathogens.

No.	Strain	MIC Value (μM)	Antibiotic Resistance
1	*E. faecalis* ATCC29212	4	-
2	*E. faecalis* ATCC51575	8	vancomycin
3	*E. faecalis* 1 ^a^	8	vancomycin
4	*E. faecalis* 2 ^a^	8	vancomycin
5	*E. faecalis* 3 ^a^	8	vancomycin
6	*E. faecalis* 4 ^a^	4	vancomycin
7	*E. faecalis* 5 ^a^	8	vancomycin
8	*E. faecalis* 6 ^a^	8	vancomycin
9	*Staphylococcus aureus* ATCC29213	>64	-
10	MRSA ATCC1717	>64	methicillin
11	*Escherichia coli* ATCC25922	>64	-
12	*Escherichia coli* O157:H7 NCTC12900	>64	-
13	*Listeria monocytogenes* ATCC19114	>64	-
14	*Salmonella* ATCC51005	>64	-
15	*Pseudomonas aeruginosa* PA01	>64	-
16	*Klebsiella pneumoniae* ATCC78578	>64	-

^a^ indicates the *E. faecalis* was isolated from food; - indicates sensitive bacteria.

## Data Availability

The original contributions presented in the study are included in the article/Supplementary Materials; further inquiries can be directed to the corresponding author.

## Supplementary Materials

The following supporting information can be downloaded at: https://www.mdpi.com/article/10.3390/foods13101448/s1, Figure S1: Effect of paracin wx7 on DNA of *E. faecalis*.
