# Supplementary material for: Antibacterial Activity and Action Mechanism of Bacteriocin Paracin wx7 as a Selective Biopreservative against Vancomycin-Resistant Enterococcus faecalis in Fresh-Cut Lettuce
Source: Foods. 2024 May 8;13(10):1448. doi: 10.3390/foods13101448 (PMC11119456; doi:10.3390/foods13101448)
Supplement: Supplementary file 1 [file foods-13-01448-s001.zip › foods-2976268-supplementary.pdf]

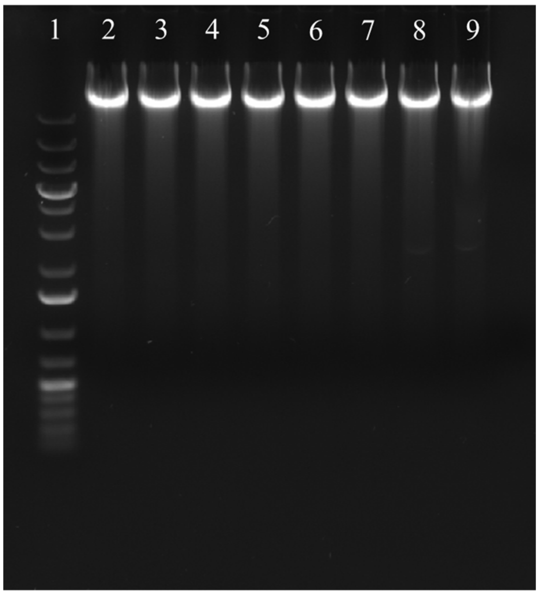

Figure. S1 Effect of paracin wx7 on DNA of *E. faecalis*.

Lane 1 is DNA marker (geneRuler 1kb plus, ThermoFisher, USA), lane 2 is DNA of *E. faecalis* without bacteriocin treatment, lane 3 to 9 are DNA of *E. faecalis* treated by paracin wx7 (DNA : bacteriocin) at ratio of 3.125:200, 6.25:200; 12.5:200; 25:200, 50:200, 100:200, 200:200, respectively (μg/mL : μg/mL).
